# A Non-Flammable Zwitterionic Ionic Liquid/Ethylene Carbonate Mixed Electrolyte for Lithium-Ion Battery with Enhanced Safety

**DOI:** 10.3390/ma14154225

**Published:** 2021-07-28

**Authors:** Zeliang Guan, Zhijun Zhang, Binyang Du, Zhangquan Peng

**Affiliations:** 1 MOE Key Laboratory of Macromolecular Synthesis and Functionalization, Department of Polymer Science & Engineering, Zhejiang University, Hangzhou 310027, China; 21929001@zju.edu.cn (Z.G.); 11529022@zju.edu.cn (Z.Z.); 2 Laboratory of Advanced Spectro-Electrochemistry and Lithium-Ion Batteries, Dalian National Laboratory for Clean Energy, Dalian Institute of Chemical Physics, Chinese Academy of Sciences, Dalian 116023, China

**Keywords:** zwitterionic ionic liquid, electrolyte, lithium-ion battery, non-flammability, excellent cycling stability

## Abstract

Today, the requirement for clean, highly efficient, and safe energy seems to be higher and higher due to non-renewable energy and pollution of the environment. At this moment, lithium-ion batteries (LIBs) look like a reliable solution for this dilemma since they have huge energy density. However, the flammability of the conventional electrolyte used in the LIBs is one of critical disadvantages of LIBs, which compromises the safety issue of LIBs. Herein, we reported a non-flammable zwitterionic ionic liquid-based electrolyte named TLPEC, which was fabricated by simply mixing a novel zwitterionic ionic liquid TLP (93 wt%) and ethylene carbonate (EC, 7 wt%). The TLPEC electrolyte exhibited a wide electrochemical potential window of 1.65–5.10 V and a robust ionic conductivity of 1.0 × 10^−3^ S cm^−1^ at 20 °C, which renders TLPEC to be a suitable electrolyte for LIBs with enhanced safety performance. The LIBs, with TLPEC as the electrolyte, exhibited an excellent performance in terms of excellent rate capability, cycling stability, and high specific capacity at 25 and 60 °C, which were attributed to the stability and high ionic conductivity of TLPEC electrolyte during cycling as well as the excellent interface compatibility of TLPEC electrolyte with lithium anode.

## 1. Introduction

Lithium-ion batteries (LIBs) have attracted extensive attention because of the urge for clean energy and less pollution to the environment. Typically, LiFePO_4_ (LFP) is used as cathode material of LIBs, which can provide a theoretical charge capacity of 170 mAh g^−1^ for LIBs [1,2], and has the advantages of high temperature stability, environmental friendliness, and rich resources [3,4,5]. Organic solvents such as propylene carbonate (PC), dimethyl carbonate (DMC), 1, 2-dimethoxyethane (DME), and tetraethylene glycol dimethylether (TEGDME) are widely used as electrolytes for LIBs because of their low melting points, high ionic conductivities, and low costs [6,7]. However, these above-mentioned common electrolytes have some disadvantages, such as low flashing point and flammability, which will lead to fire if local overheating in LIBs is encountered. The flammability of electrolytes is the main origin of safety issue for LIBs. To develop new kinds of electrolytes that are nonflammable and exhibit excellent electrochemical properties is an important task that has attracted extensive investigations in the scientific community.

To solve the flammability issue of electrolytes and improve the safety of LIBs, many kinds of electrolytes have been explored, such as polymeric electrolytes, solid-state electrolytes, and ionic liquid electrolytes [8,9,10,11]. Among them, ionic liquid electrolytes seem to be more competitive because of their non-flammability and wide operative temperature range [12,13,14]. Room temperature ionic liquid (RTIL) is usually constituted of an organic cation (e.g., imidazolium, sulfonium, morpholinium, phosphonium, pyrrolidinium, boronium, etc.) and an inorganic/organic anion (e.g., TFSI^−^, PF_6_^−^, FSI^−^, etc.) [15,16,17]. The RTILs have negligible volatility, low melting point (as low as −80 °C), wide electrochemical window (0–5 V vs. Li^+^/Li), and high ionic conductivity (up to 0.1 S cm^−^^1^) [18]. More importantly, the RTILs are nonflammable [19,20,21]. Therefore, RTILs are excellent candidates as the electrolytes for solving the safety issue of LIBs. For example, Menne et al. [22] reported an ionic liquid-based electrolyte, which consisted of the RTIL, triethylammonium bis (tetrafluoromethylsulfonyl) amide and 1 M LiTFSI, for the LIBs with LFP and Li_4_Ti_5_O_12_ (LTO) as cathode and anode, respectively. The discharge capacities of these LIBs were 115, 60, and 30 mAh g^−1^, respectively, when operating at 0.1, 1, and 5 C. Le et al. [23] developed an electrolyte made by mixing 80 vol% ionic liquid namely 1-ethyl-3-methylimidazolium bis (trifluoromethanesulfonul) imide (EMITFSI), 20 vol% ethylene carbonate (EC), and 0.25 M LiTFSI. By mixing with 20 vol% EC, the conductivity of EMITFSI increased from 9.10 × 10^−3^ to 15.8 × 10^−3^ S cm^−1^. The LIBs using this mixed electrolyte exhibited a specific capacity of 130 mAh g^−1^ and 3% capacity loss within 30 cycles under a charge and discharge current density of 0.1 C. However, the charge–discharge capacity and rate capability of these LIBs with IL-based electrolytes required further improvement. To develop efficient IL-based electrolytes, which can be used in practices for achieving safe and long cycle lift LIBs, is still a challenged task.

**Scheme 1:**
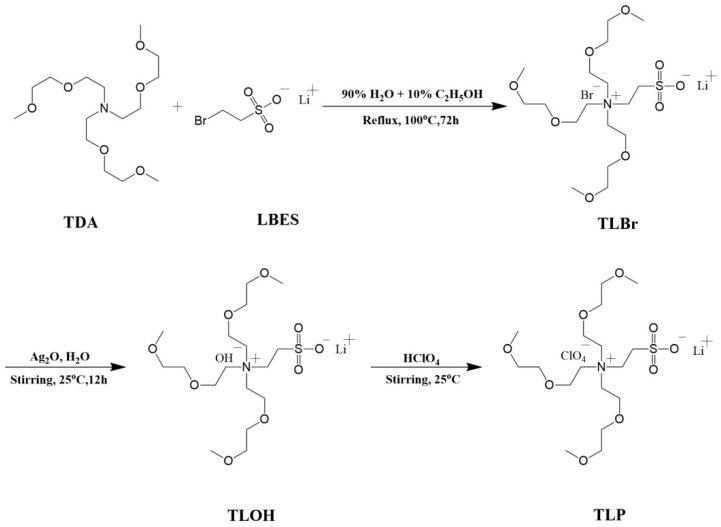
Synthesis routine of TLP.

In the present work, we shall report a kind of zwitterionic ionic liquid coded as TLP, which was obtained by first quaternizing tris(dioxa-3,6-heptyl)anime (TDA) with lithium 2-bromoethanesulphoate (LBES) and then performing the anion exchange reaction against perchloric acid (HClO_4_) (Scheme 1). The zwitterionic IL has the cation and anion in one molecule and two ion pairs of positive and negative ions [24]. In such an zwitterionic ILs system, the mobile anion and cation are in equal stoichiometric ratio. As a result, the mobility of counterpart ions is thus inhibited, leading to higher Li^+^ conductivity [25,26]. Such zwitterionic IL, TLP, was proved to be an efficient component for constructing electrolytes with improved safety like TLPEC for LIBs, which can be operated at a wide temperature range. An optimal mixed electrolyte named as TLPEC was made by simply mixing 93 wt% TLP with 7 wt% EC. No additional lithium salt was required because TLP carried the Li^+^ ion itself. The obtained TLPEC was non-flammable and had high ionic conductivity and a wide electrochemical potential window. It shall be shown that the LSBs with TLPEC as the electrolyte exhibited a high specific capacity with good capacity retention and cycling stability at 25 and 60 °C.

## 2. Materials and Methods

### 2.1. Materials

Tris(dioxa-3,6-heptyl)anime (TDA, 95%), sodium 2-bromoethanesulphoate (SBES, 98.5%), lithium hydroxide (LiOH, 99%), and silver(I) oxide (Ag_2_O, 99%) were purchased from J&K Chemical Ltd, Shanghai, China. Perchloric acid (HClO_4_, 70%) and ethylene carbonate (EC, 99%) were purchased from Aladdin Chemical Ltd, Shanghai, China. All of the chemicals were used as received.

### 2.2. Fabrication of Lithium 2-Bromoethanesulphoate (LBES)

The cation exchange technology was used to prepare the LBES. A total of 5 mL SBES (2.00 g, 9.47 mmol) aqueous solution was passed through the cation exchange resin by using deionized water as eluent until the effluent droplet was neutral. Then, 0.226 g LiOH (9.47 mmol) was added into the obtained solution under stirring. After 5 min, rotary evaporation was performed to get rid of water, leading to the targeted white solid LBES (1.73 g, 8.87 mmol).

### 2.3. Synthesis of TLP

TLP was synthesized as follows: 4.54 g LBES and 8.28 g TDA were added in the 30 mL water/ethanol (9/1) mixed solvent and refluxed at 100 °C. After 72 h, the solvent was removed by rotary evaporation. Then, 20 mL dichloromethane and 20 mL deionized water were added. The collected aqueous phase was extracted again by using 20 mL dichloromethane. After drying the aqueous phase, 10.55 g liquid products were obtained and named as TLBr. 4.72 g Ag_2_O and 10 mL deionized water was then added into TLBr under stirring for 12 h. The precipitate was removed by filtration. Afterward, 1.652 mL HClO_4_ solution (20.35 mmol) was further added and stirred for 12 h. Finally, water was removed by rotary evaporation, leading to 8.72 g targeted zwitterionic ionic liquid TLP.

### 2.4. Preparation of LiFePO_4_ Cathode and Zwitterionic Ionic Liquid Based Electrolyte

The LiFePO_4_ cathode was obtained by mixing poly(vinylidence fluoride) (PVDF, Kynar HSV900, ARKEMA, Pairs, France), carbon black (SUPER P Li, TIMCAL Ltd., Bodio, Switzerland), and LiFePO_4_ (Shanghai Macklin Biochemical Co., Ltd., Shanghai, China) with a weight ratio of 1:1:8 and aluminum foil (Guangdong Canrd New Energy Technology Co., Ltd., Dongguan, China) as the current collector. The LiFePO_4_ cathode was dried at 80 °C under vacuum for 24 h and then placed in the glove-box before use.

The zwitterionic ionic liquid-based electrolyte named as TLPEC was prepared by simply mixing TLP with small amount of EC. The final concentration of EC in TLPEC electrolyte was about 7 wt%.

### 2.5. Physical Characterizations

The ^1^H-NMR spectrum of TLP was obtained by using a Bruker 400 MHz spectrometer (Bruker Corp., Karlsruhe, Germany) with D_2_O as a solvent. The phase transition temperature of TLP was measured by using a Q20 differential scanning calorimeter (DSC, TA Instrument, Inc., New Castle, PA, USA) with a ramping rate of 10 °C/min from −80 to 50 °C in nitrogen atmosphere. TLP was first heated to 50 °C and then quickly cooled down to −80 °C. The second heating process was performed from −80 to 50 °C to determine the transition temperature of TLP. The inflection point (the maximum slope point) was determined as the transition temperature of TLP from its DSC curve. The thermal stability of TLP was investigated by using a Q50 thermogravimetric analysis (TGA, TA Instrument, Inc., New Castle, PA, USA) instrument with a heating rate of 10 °C/min in N_2_ atmosphere. The temperature (T_5%_) with the sample mass loss of 5% was determined to be the decomposition temperature of TLP. Three to five milligrams of TLP were used for DSC and TGA measurements, respectively. TLP was measured after heating at 70 °C under vacuum over 24 h to remove the water. Scanning electron microscopy (SEM) was used to observe the surface morphology of lithium metal by using a Hitachi S-4800 SEM, (Hitachi, Ltd., Tokyo, Japan).

### 2.6. Electrochemical Characterization

The CR2032-type coin cells assembled in an Ar-filled glovebox were used for the electrochemical characterization by using Bio-logic VMP3 multichannel potenetiostatic–galvanostatic system (BioLogic SAS, Seyssinet-Pariset, France). The 4 Å molecular sieves were put into TLPEC over 24 hours to remove the trace water in ILs so that it could be used as electrolyte in LIBs. For each cell, 100 μL TLPEC was used. The charge–discharge cycling tests of LIBs with TLPEC electrolyte (CR2032-type coin cells) were characterized by using a LAND battery testing system (CT2001A, Wuhan LAND Electronic Co.Ltd., Wuhan, China). CR2032 coin cell with stainless steel electrodes were used to measure the conductivity (σ) of TLPEC. The impedance of the [stainless steel anode |TLPEC| stainless steel cathode] coil cell was measured in the frequency range of 100 mHz–100 kHz with the perturbation amplitude of 5 mV at various temperatures. The ionic conductivities were given as, σ = L/(R × A), where L is the thickness of the separator, R is the resistance of cell, and A is the area of the separator. Linear sweep voltammetry measurements were carried out with the open-circuit voltage of 5.5–1.0 V vs. Li/Li^+^ and a potential scan rate of 1 mV s^−1^ to determine the electrochemical stabilization window of TLPEC. The Li^+^ transfer number of TLPEC electrolyte was measured with a symmetric [Li metal anode |TLPEC electrolyte| Li metal cathode] coil cell under a polarization voltage of 10 mV for 8 h. The impedance of the [Li metal anode | TLPEC electrolyte | Li metal cathode] coil cell was measured in the frequency range of 100 mHz–100 kHz with the perturbation amplitude of 5 mV.

## 3. Results and Discussion

**Figure 1:**
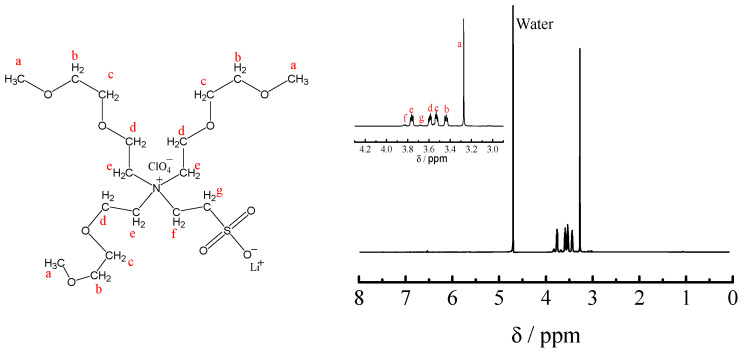
^1^H-NMR spectrum of TLP in D_2_O.

**Figure 2:**
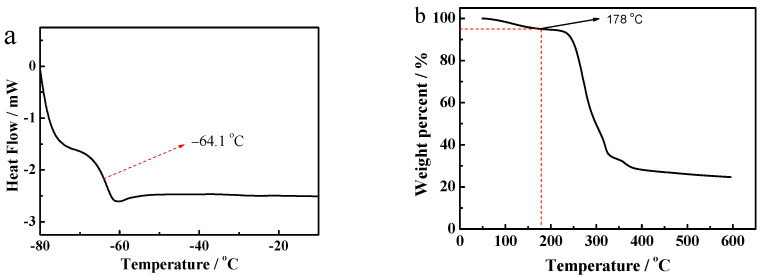
(**a**) DSC and (**b**) TGA data curves of TLP.

TLP, was synthesized by first quaternizing tris(dioxa-3,6-heptyl)anime (TDA) with lithium 2-bromoethanesulphoate (LBES) and then performing the anion exchange reaction against perchloric acid (HClO_4_). Figure 1 shows the ^1^H-NMR spectrum of TLP, which confirms the chemical structure of TLP. The chemical shifts of peaks are assigned as follows: 3.27 ppm (s, 9H, CH_2_-CH_2_-OCH_3_), 3.44 ppm (t, 6H, CH_2_-CH_2_-OCH_3_), 3.53 ppm (m, 6H, CH_2_-CH_2_-OCH_3_), 3.59 ppm (m, 6H, N^+^-CH_2_-CH_2_), 3.68 ppm (m, 2H, CH_2_-CH_2_-SO_3_^-^), 3.76 ppm (t, 6H, N^+^-CH_2_-CH_2_), and 3.83 ppm (m, 2H, CH_2_-CH_2_-SO_3_^-^). The obtained TLP is a liquid at room temperature. Figure 2a,b show the DSC and TGA data curves of TLP, respectively, which indicate that TLP has a glass transition temperature (*T*_g_) of about −64.1 °C and a thermal decomposition temperature of about 178 °C. The low *T*_g_ and high decomposition temperature of TLP indicate that TLP can be potentially used as an electrolyte component for LIBs. However, for the preliminary experiments using pure TLP as the electrolyte for LIBs, the obtained [Li metal anode |TLP| LiFePO_4_-based cathode] battery only exhibited a highest specific capacity of 55 mAh g^−1^, which quickly decayed to 0.5 mAh g^−1^ within 500 cycles at a charge–discharge current density of 0.5 C under 25 °C (data not shown). These results indicated that pure TLP is not suitable to be used solely as the electrolyte for LIBs. The addition of other electrolytes might be needed to improve the performance of TLP.

**Figure 3:**
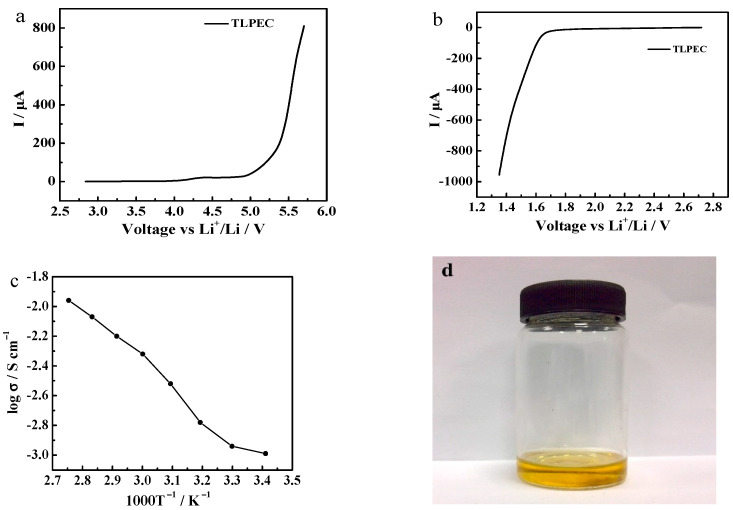
(**a**) LSV curve of TLPEC from 2.8 V to 5.6 V. (**b**) LSV curve of TLPEC from 2.8 V to 1.4 V. (**c**) Ionic conductivities of TLPEC as a function of temperature. (**d**) The photo of TLPEC.

**Figure 4:**
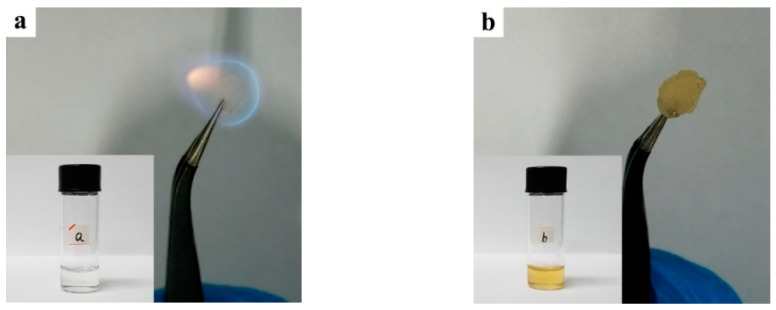
Photos of (**a**) the lighted degreased cotton that was dipped into the PC/EC (1:1) electrolyte; (**b**) the degreased cotton that was dipped into the TLPEC electrolyte and lit with a lighter.

Ethylene carbonate (EC) is chosen as an additive electrolyte for TLP in the present work because EC is solid at room temperature with a melting point of 38 °C and hence is less flammable. EC cannot solely be used as the electrolyte for LIBs. However, the mixed electrolytes of EC with PC or DEC are excellent electrolytes for LIBs. The performance of LIBs with mixed electrolytes, which were prepared by mixing TLP with 3%, 5%, 7%, and 10% EC, respectively, were preliminarily screened. An optimal mixed electrolyte, named as TLPEC, was thus obtained by mixing 93 wt% TLP and 7 wt% EC. The amount of EC in the mixed electrolyte was controlled to be as less as possible so that the safety performance of zwitterionic ionic liquid, TLP would not be compromised. Note that no additional lithium salt was added because TLP contained counter-cation Li^+^ and counter-anion ClO_4_^−^ itself. Figure 3a,b shows that TLPEC was stable in the voltage range of 1.65–5.10 V at 25 °C, which was wider than the cutoff voltage of LIBs with LFP as the cathode, i.e., 2.7 to 3.8 V. Figure 3c shows that TLPEC exhibited an ionic conductivity of 1.0 × 10^−3^ S cm^−1^ at 20 °C, which increased to 1.1 × 10^−2^ S cm^−1^ at 90 °C. Figure 3d shows the photograph of TLPEC, showing its liquid feature. The ignition experiments were further carried out to testify to the flammability of TLPEC. Mixed electrolyte of PC/EC with a weight ratio of 1:1 was also prepared for comparison. Degreased cottons were then dipped into PC/EC and TLPEC electrolytes, respectively, which were then ignited with a lighter. Figure 4 and Videos S1 and S2 show the lighting results of ignition experiments, which indicated that after dipping into PC/EC mixed electrolyte, the degreased cotton could be easily lit (Video S1). However, the degreased cotton that was dipped into TLPEC electrolyte can hardly be lit (Video S2). These results confirmed that the traditional PC/EC mixed electrolyte was easily ignited as expected and the TLPEC electrolyte was non-flammable. Therefore, both the high ionic conductivity and the wide electrochemical window might render TLPEC to be a suitable electrolyte for LIBs, and the non-flammability character of TLPEC would enhance the safety performance of the corresponding LIBs.

**Figure 5:**
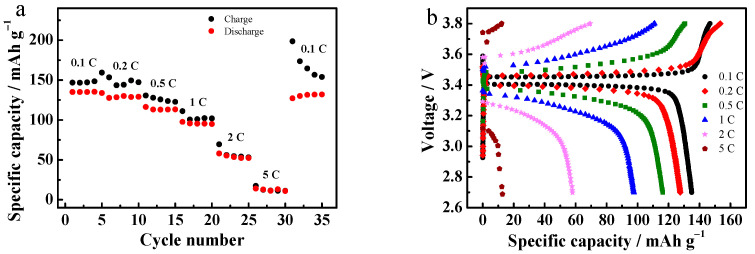
(**a**) Rate performance of the LIBs with TLPEC electrolyte measured with various charge and discharge current densities at 25 °C. (**b**) The first cycle of charge and discharge curves of the LIBs with TLPEC electrolyte under various current densities at 25 °C.

The rate capabilities of the obtained LIBs with TLPEC electrolyte at 25 °C were then studied. Figure 5a shows that the specific charge/discharge capacity of the LIBs with TLPEC electrolyte under various charge and discharge current densities from 0.1 to 5 C. It can be seen that the specific capacity of the LIBs gradually decreased from about 135 mAh g^−1^ at 0.1 C to about 13 mAh g^−1^ at 5 C and increased back to about 131 mAh g^−1^ when the current density was switched back to 0.1 C. The specific capacity of LIBs dropped sharply at current density of 5 C, which might suggest that the Li^+^ ions in TLPEC electrolyte had lower mobility at high charge and discharge current density. The Li^+^ transfer number (*t*_Li+_) of TLPEC electrolytes was measured to be 0.14 at 25 °C. The value of *t*_Li+_ can reflect the mobility of Li^+^ ions in LIBs. Such low *t*_Li+_ might be due to the fact that the quaternized cations also transfer during the discharge process of LIBs, resulting in the decrease of *t*_Li+_. The low *t*_Li+_ of TLPEC electrolytes thus accounted for the significant decrease of specific capacity at 5 C. Figure 5b shows the first cycle charge and discharge curves of the LIBs with TLPEC electrolyte at different charge and discharge current densities. At low current density of 0.1 C, the LIBs had high specific capacity and the charge/discharge platforms can be obviously observed. These results indicated that the internal resistance and polarization voltage of battery were small at low current densities. With increasing the current density, the specific capacity of LIBs continuously decreased and the charge/discharge platforms gradually disappeared, indicating that the polarization voltage increased rapidly and the internal resistance of the battery became larger at higher current density.

**Figure 6:**
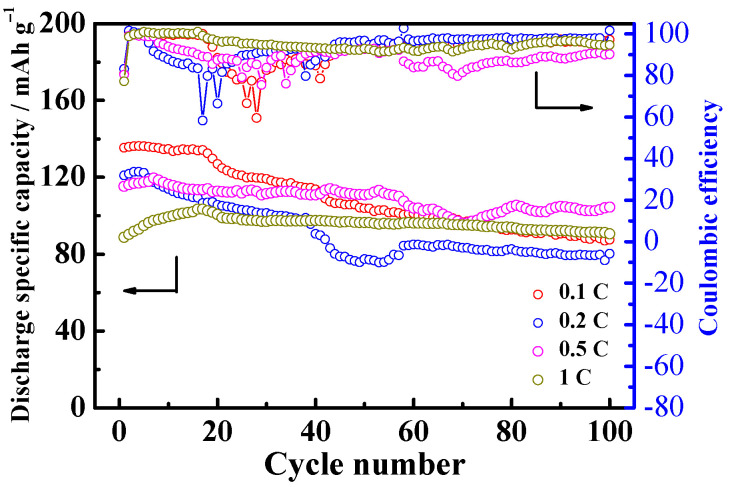
Cycling stabilities of LIBs with TLPEC electrolyte under different charge and discharge current densities at 25 °C.

Figure 6 shows the cycling performance of the obtained LIBs with TLPEC electrolyte under various current densities at 25 °C. The specific capacities of LIBs with current densities of 0.1, 0.2, 0.5, and 1 C were 135.4, 120.9, 115.3, and 88.8 mAh g^−1^ at the first cycle, respectively. After 100 cycles, the corresponding specific capacities decayed to be 87.5, 80.2, 104.5, and 90.5 mAh g^−1^, respectively. The specific capacities of the LIBs at the first cycle decreased with increasing current density, which was mainly attributed to the increase of polarization inside the batteries. After 100 cycles, the retentions of specific capacities were about 72.0%, 66.8%, 90.9%, and 101.9% for the LIBs operated with 0.1, 0.2, 0.5, and 1 C, respectively. Furthermore, the Coulombic efficiency of LIBs fluctuated before 50 cycles with low current densities like 0.1 and 0.2 C. Possibly, such fluctuation of Coulombic efficiency was related to the SEI film on the lithium metal surface. With low current densities, the SEI film might not be completely formed or reach a stable state at an earlier time. The SEI film might be formed quickly with higher current density. As a result, Coulombic efficiency of LIBs had less fluctuation with the current densities of 0.5 and 1 C.

**Figure 7:**
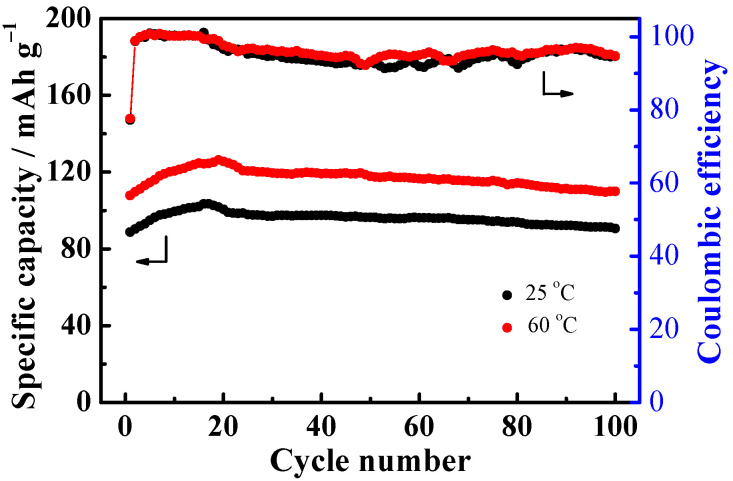
Cycling performances of the LIBs with TLPEC electrolyte at 25 and 60 °C. The charge and discharge current density was 1 C.

It can be seen from Figure 3 and Figure 4 that the TLPEC electrolyte was non-flammable and its ionic conductivity increased with increasing temperature. The performance of LIBs with TLPEC electrolyte was further investigated at high temperature, i.e., 60 °C. Figure 7 shows the long-term cyclic performances of LIBs with TLPEC electrolyte at 60 °C with current density of 1 C. Note that the cyclic performances of the corresponding LIBs at 25 °C was also included for comparison. The discharge specific capacity of LIBs with TLPEC electrolyte was about 107.8 mAh g^−1^ at the first cycle at 60 °C. During cycling, the discharge specific capacity even increased up to 126.3 mAh g^−1^ at the 19th cycle. The discharge specific capacity decayed to be 109.4 mAh g^−1^ after 100 cycles. The capacity retention was about 86.6% when referring to the highest value of 126.3 mAh g^−1^. If referring to the value of 107.8 mAh g^−1^ at the first cycle, a value of 101.5% was obtained for the capacity retention after 100 cycles. The overall cyclic performance and specific capacity of the LIBs with TLPEC electrolyte at 60 °C and 1 C were superior to those of the LIBs with TLPEC electrolyte at 25 °C. These results might suggest the excellent electrochemical performance of LIBs with TLPEC electrolyte at high-temperature. As shown in Table 1, compared with the other ionic liquid-based electrolytes reported in the literature, the cycling performance of LIBs with TLPEC electrolyte was improved even at higher current density and low operated temperature.

**Figure 8:**
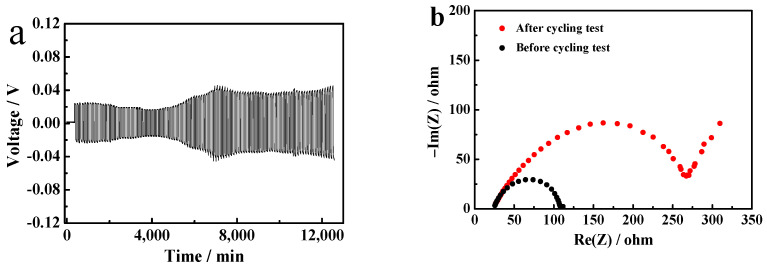
(**a**) The charge and discharge cycling measurement and (**b**) EIS results of Li | Li symmetrical cell with TLPEC electrolyte: constant current (0.1 mA cm^−2^) for 60 min; 100 cycles at 25 °C.

The charge–discharge cycling test of symmetrical cell with lithium metal electrodes and TLPEC electrolyte at 25 °C was carried out to investigate the compatibility of TLPEC electrolyte with Li metal anode. Figure 8a shows that the polarization voltage first slightly decreased and then increased to a stable platform during the cycling measurement. The initial fluctuation of polarization voltage indicated the initial formation and growth of the SEI film, which was consistent with the fluctuation of Coulombic efficiency observed at the early 50 cycles with low current densities as shown in Figure 6. When the uniform homogeneous SEI film was formed, the polarization voltage of the lithium symmetrical cell with the TLPEC electrolyte was stabilized. The impedance spectra of the lithium symmetrical cell before and after running for 100 cycles are shown in Figure 8b. The bulk resistance (*R*_b_) of TLPEC electrolyte was almost unchanged after 100 cycles, *R*_b_s before and after cycling test were 23.88 and 25.56 Ω, respectively. The obvious increase of the semicircle after the cycling test meant a larger interfacial resistance (*R*_i_). *R*_i_ increased from 69.4 Ω to 252.7 Ω after cycling measurement, indicating the formation of an SEI film on the lithium surface. These results indicated that the TLPEC electrolyte had excellent compatibility with lithium metal.

**Figure 9:**
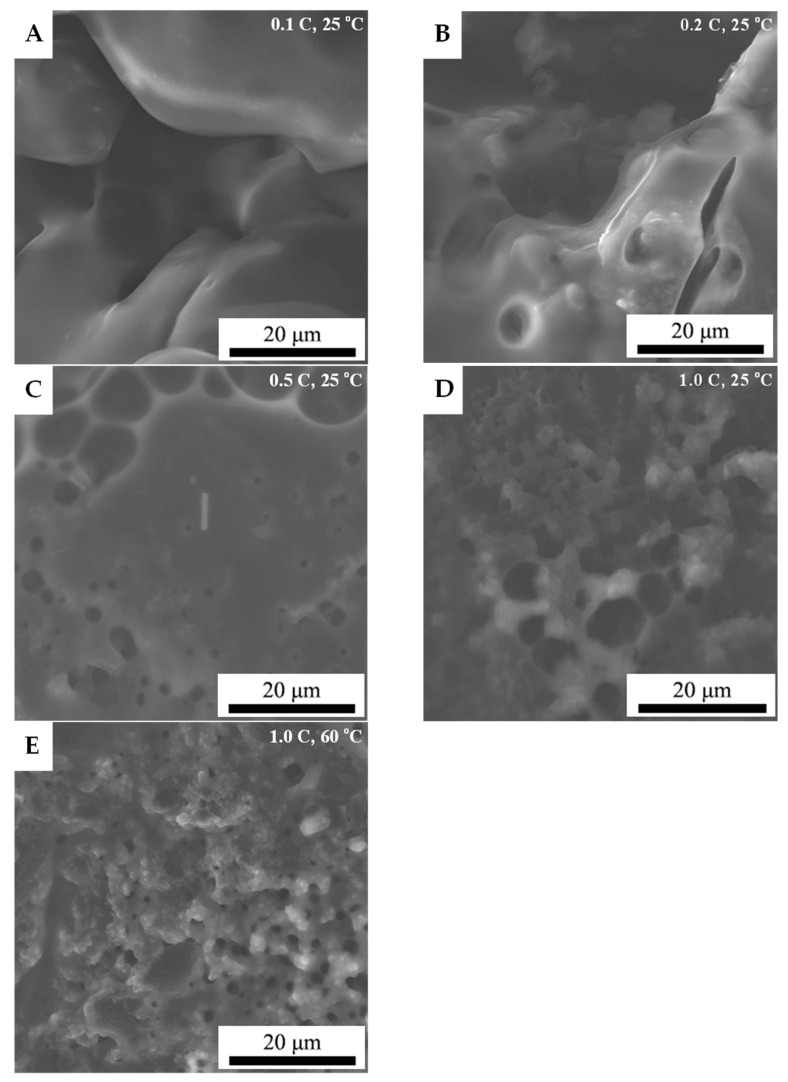
The SEM morphologies of the lithium anodes of [Li metal anode | TLPEC electrolyte | LiFePO_4_-based cathode] batteries after 100 charge–discharge cycles at various current densities and temperatures: (**A**) 0.1 C at 25 °C; (**B**) 0.2 C at 25 °C; (**C**) 0.5 C at 25 °C; (**D**) 1 C at 25 °C; and (**E**) 1 C at 60 °C.

To explore the relationship between the cycling performance of LIBs with TLPEC as the electrolyte and the SEI film formed on the lithium surface during cycling, the LIBs after 100 cycles under various charge/discharge current densities and temperatures were disassembled. The surface morphologies of the corresponding lithium anodes were then observed by SEM, as shown in Figure 9. By comparing Figure 9A−D, a thin layer of porous SEI film was observed on the lithium surface after 100 cycles at a large current density of 1 C. However, with the increase of charging/discharging current, the pore size of lithium dendrite increased gradually and local cracks appeared, resulting in the uneven deposition of lithium dendrite. Furthermore, the surface of lithium metal was more uniform and the pore size of SEI film was smaller at 60 °C, as shown in Figure 9E. It can be concluded from the results of Figure 6 and Figure 7 that the smaller and more uniform the pore size of SEI film on the lithium surface was, the more stable the LIBs with TLPEC electrolyte were during charge/discharge cycling measurement. The formation of porous and homogeneous SEI film on the lithium surface thus improved the cycle stability of the LIBs with TLPEC electrolyte.

## 4. Conclusions

A novel zwitterionic ionic liquid named TLP was fabricated, which has a low *T*_g_ of −64.1 °C and a high thermal decomposition temperature of about 178 °C. An optimal electrolyte, TLPEC, was prepared by simply mixing 93 wt% TLP with 7 wt% EC, which was non-flammable and exhibited a robust ionic conductivity of 1.0 × 10^−3^ S cm^−1^ at 20 °C and a wide electrochemical potential window of 1.65–5.10 V. Such non-flammable TLPEC electrolyte can be applied as the electrolyte for LIBs with enhanced safety performance, which exhibited excellent performance in terms of excellent cycling stability, excellent rate capability, and high capacity at 25 and 60 °C. A specific capacity of 135.4 mAh g^−1^ was achieved at 0.1 C and 25 °C for LIBs with TLPEC electrolyte, whereas a specific capacity of 107.8 mAh g^−1^ was obtained at 1 C and 60 °C. The TLPEC electrolyte was proved to have good compatibility with lithium metal by forming a stable SEI film.

## Figures and Tables

**Table 1:** Discharge specific capacities of LIBs with various ionic liquid-based electrolytes reported in the literature.

Electrolyte System	C-Rate	Cycle Number	Temperature/ °C	Discharge Specific Capacity/mAh g^−^^1^
C_3_mpyrTFSI, 0.5 mol kg^−1^ LiTFSI, and 0.25 mol kg^−1^ SMKTFSI	0.267	100	50	130 [27]
[N_111_N_112_BH_2_][TFSI] and 0.5 mol kg^−1^ LiTFSI	0.1	100	20	100 [17]
1,2-DMImTFSI and 0.5 M LiTFSI	1	100	60	100 [28]
[C_1_C_1_C_6_Im][Li][NTf_2_] and 5 vol% VC	0.1	30	60	120 [29]
0.9/0.1 molar ratio for P_1,2O2_TFSI/LiTFSI and 10 w/w% EC:PC:DMC (1:1:3 w/w%)	0.2	30	-	90 [15]
[Li][C_1_C_3_CNIm][NTf_2_] and 2%VC	0.1	125	60	50 [30]
Li_0.2_Pyr13_0.8_TFSI	0.2	100	80	120 [31]
93 wt% TLP and 7 wt% EC (this work)	0.5	100	25	120

## Data Availability

Data is contained within the article and Supplementary Materials.

## Supplementary Materials

The following are available online at https://www.mdpi.com/article/10.3390/ma14154225/s1, Video S1: ignition experiment of degreased cotton after dipping into PC/EC mixed electrolyte, Video S2: ignition experiment of degreased cotton after dipping into TLPEC electrolyte.
